# The RNA-binding protein hnRNPLL induces a T cell alternative splicing program delineated by differential intron retention in polyadenylated RNA

**DOI:** 10.1186/gb-2014-15-1-r26

**Published:** 2014-01-29

**Authors:** Vicky Cho, Yan Mei, Arleen Sanny, Stephanie Chan, Anselm Enders, Edward M Bertram, Andy Tan, Christopher C Goodnow, T Daniel Andrews

**Affiliations:** 1 Immunogenomics Laboratory, John Curtin School of Medical Research, Australian National University, GPO Box 334, Canberra City ACT 2601, Australia; 2 Immunology Groups, Bioprocessing Technology Institute, 20 Biopolis Way, Centros, 138668 Singapore, Singapore; 3 Microarray Groups, Bioprocessing Technology Institute, 20 Biopolis Way, Centros, 138668 Singapore, Singapore; 4 Ramaciotti Immunisation Genomics Laboratory, John Curtin School of Medical Research, Australian National University, GPO Box 334, Canberra City ACT 2601, Australia; 5 Australian Phenomics Facility, Hugh Ennor Building, Australian National University, Garran Road, ACT 0200 Canberra, Australia

## Abstract

**Background:**

Retention of a subset of introns in spliced polyadenylated mRNA is emerging as a frequent, unexplained finding from RNA deep sequencing in mammalian cells.

**Results:**

Here we analyze intron retention in T lymphocytes by deep sequencing polyadenylated RNA. We show a developmentally regulated RNA-binding protein, hnRNPLL, induces retention of specific introns by sequencing RNA from T cells with an inactivating *Hnrpll* mutation and from B lymphocytes that physiologically downregulate *Hnrpll* during their differentiation. In *Ptprc* mRNA encoding the tyrosine phosphatase CD45, hnRNPLL induces selective retention of introns flanking exons 4 to 6; these correspond to the cassette exons containing hnRNPLL binding sites that are skipped in cells with normal, but not mutant or low, hnRNPLL. We identify similar patterns of hnRNPLL-induced differential intron retention flanking alternative exons in 14 other genes, representing novel elements of the hnRNPLL-induced splicing program in T cells. Retroviral expression of a normally spliced cDNA for one of these targets, *Senp2*, partially corrects the survival defect of *Hnrpll*-mutant T cells. We find that integrating a number of computational methods to detect genes with differentially retained introns provides a strategy to enrich for alternatively spliced exons in mammalian RNA-seq data, when complemented by RNA-seq analysis of purified cells with experimentally perturbed RNA-binding proteins.

**Conclusions:**

Our findings demonstrate that intron retention in mRNA is induced by specific RNA-binding proteins and suggest a biological significance for this process in marking exons that are poised for alternative splicing.

## Background

Splicing of introns from pre-messenger RNA is a tightly regulated process performed by the spliceosome [1]. The assembly of the spliceosome at appropriate exon-intron boundaries is influenced by regulatory RNA-binding proteins (RBPs) that operate antagonistically to both enhance and repress this splicing machinery [2-4]. Heterogeneous nuclear ribonucleoproteins (hnRNPs) predominantly perform a splicing repressor function and their binding blocks spliceosome assembly and leads to exclusion of exons from mature mRNAs. Serine/arginine-rich (SR) proteins bind enhancer sequences in RNA and promote association of spliceosomal proteins at splice sites. This general model nevertheless requires testing in physiological experimental systems where developmentally regulated splicing factors are specifically perturbed, but few such systems have been described, especially for mammalian cells.

Recent information has identified that transcription and splicing are functionally coupled, allowing cross-talk between the two processes [5]. Co-transcriptional assembly of the spliceosome on nascent, chromatin-associated RNA transcripts is a significant factor in correct gene regulation in yeast [6], *Drosophila*[7] and mammals [8-10]. The spliceosome has been shown to quickly associate with chromatin-associated nascent RNA in human HeLa cells [11] and slowing the rate of RNA polymerase II transcription in *Drosophila* greatly increases the rate of alternative exon inclusion [12]. High-throughput sequencing of chromatin-associated, nascent RNA in *Drosophila* has revealed that the majority of introns are co-transcriptionally spliced at least half of the time, though a minority of introns are spliced slowly and some appear never to be co-transcriptionally spliced [7]. This variability in co-transcriptional splicing efficiency occurs even within single transcripts and suggests that splicing is regulated at the level of the intron [7], presumably by different RBPs such as the hnRNPs and SR proteins. Introns that are consistently identified to be resistant to co-transcriptional splicing correlate with annotated alternative exons [7,9,13].

To understand mammalian alternative splicing, and define the relationship between variable intron retention after transcription and alternative splicing, it would be useful to be able to experimentally perturb developmentally regulated alternative splicing events through genetic mutations in the specific RBPs that control them. One of the best defined mammalian alternative splicing events occurs in the *Ptprc* gene encoding the major plasma membrane tyrosine phosphatase, CD45, in T lymphocytes and other blood leukocytes [4,14]. In memory T cells that have been activated previously by antigens, exons 4, 5 and 6 are skipped in the translated *Ptprc* mRNA. The resulting loss of the CD45-RA, RB and RC domains in the extracellular domain of the protein, detected by flow cytometric staining with specific antibodies, is used as the primary marker to differentiate memory T cells and activated T cells (CD45-RO^+^) from naïve T cells (CD45-RA^+^ or CD45-RB^+^). Even in naïve T cell mRNA all three *Ptprc* cassette exons are rarely included whereas they are all included in B lymphocyte *Ptprc* mRNA, resulting in the CD45R-ABC isoform (B220) that is detected by specific monoclonal antibodies to identify B cells.

Silencing of *Ptprc* exons 4, 5 and 6 in T cells requires hnRNPLL, a protein with three RNA-recognition motif (RRM) domains whose mRNA expression correlates with *Ptprc* exon exclusion: it is highest in CD45RO^+^ activated and memory T cells that exclude exons 4 to 6, at intermediate levels in CD45RB^+^ naïve T cells, and at very low levels in CD45RABC^+^ B cells that include all three exons [15-17]. Mice homozygous for a destabilizing point mutation in the amino-terminal RRM domain, *Hrnpll*^thu^, fail to exclude exons 4, 5, and 6 in T-cell *Ptprc* mRNA and expression of CD45-RA and CD45-RC protein isoforms are increased 50-fold on different T-cell subsets [16]. Likewise, increased inclusion of *Ptprc* exons 4 to 6 occurs when hnRNPLL is depleted from human T cells by short hairpin RNA (shRNA) expression, while silencing of *Ptprc* exon 4 is induced in human T cells transfected to overexpress *Hnrpll* cDNA [15,17]. The isolated amino-terminal RRM domain normally binds with sequence specificity and micromolar affinity [16] to an RNA consensus sequence, the activation response sequence (ARS), which mediates exon silencing in activated T cells and occurs in each of *Ptprc* exons 4, 5 and 6 [18]. Thus, hnRNPLL is a developmentally regulated *Ptprc* splicing silencer whose expression and activity are critical for the regulated changes in CD45 isoforms on T and B lymphocytes.

A closely related protein, hnRNPL, has also been shown to bind *Ptprc* ARS RNA sequences present in exons 4 to 6 [19,20]. T cells from mice homozygous for a knockout of the *Hnrpl* gene have moderately increased inclusion of exons 4 and 6, resulting in a four-fold increase in CD45RA expression [21]; compared with a 50-fold increase caused by *Hnrpll* mutation. Thus, hnRNPL and hnRNPLL both contribute to exon silencing but their coordinated actions are only partly understood [4].

The interphase lifespan of *Hrnpll*^thu^ homozygous T cells is greatly shortened, resulting in decreased numbers of naïve T cells in the circulation [16]. This effect of hnRNPLL deficiency occurs even in T cells with a null *Ptprc* gene [22], indicating that hnRNPLL controls other genes contributing to T cell persistence that have yet to be identified. Here we use this mammalian system to analyze the consequences of perturbing hnRNPLL either by mutation or natural expression differences, as revealed by global mRNA changes measured by RNA-seq. hnRNPLL was required to induce a distinct pattern of intron retention surrounding its known target cassette exons in *Ptprc*. Differential retention of introns in deeply sequenced RNA provided a signature that could be used to identify other mRNAs requiring hnRNPLL for correct splicing in T cells, and this represents a general, albeit not completely specific, strategy to annotate the transcriptome for exons that are likely to undergo differential splicing.

## Results

### Alternative introns are differentially retained in Ptprc transcripts

In a mouse genetic screen we previously identified hnRNPLL as an essential regulatory factor responsible for skipping of *Ptprc* exons 3, 4 and 5 in T cells. *Thunder* mice have a loss-of-function point mutation in the *Hnrpll* gene that destabilizes the amino-terminal ARS-binding RRM domain of hnRNPLL [16]. To analyze the consequences of *Hnrpll*^*thu*^ upon T-cell mRNA splicing, we performed RNA-seq on mRNA isolated from CD8^+^ T lymphocytes purified from transgenic OT-1 T-cell receptor (TCR) mice that were either wild type or homozygous for *Hrnpll*^*thu*^. The OT-1 TCR transgenes possessed by this mouse provided a uniform T cell antigen receptor on the CD8^+^ T cells with specificity for a known ovalbumin-derived peptide and ensured that most of the cells were antigenically naïve. This resulted in comparable frequencies of naïve and memory T cells in the *Hrnpll*^*thu*^ animals and the wild-type controls, enabling the analysis to focus on the primary effects of the mutation on splicing and minimize the contribution of *Hnrpll*-independent differences in mRNA splicing between naïve and memory T cells.

**Figure 1:**
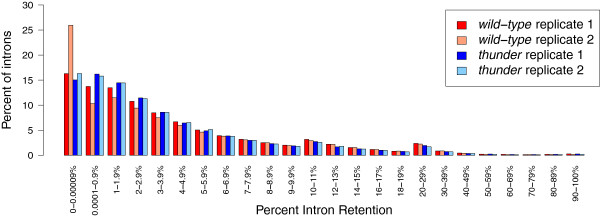
**Most transcripts in wild-type and *thunder *CD8**^**+ **^**T cells are fully spliced, yet a minority retain one or more introns.** The genome-wide distribution of intron splicing frequency is skewed towards full removal (0% intron retention), but also includes fully unspliced transcripts (100% intron retention). The majority of introns (87%) have a splicing efficiency of greater than 90%, meaning they remain unspliced in fewer than 10% of polyadenylated mRNAs; 0.94% of introns have a splicing efficiency of less than 50% and were retained in more than 50% of polyadenylated mRNAs present in our RNA-seq data.

RNA-seq was performed on biologically independent replicate samples starting with RNA purified from T cells and using oligo-dT to prime first strand cDNA synthesis. The resulting libraries were each sequenced to a depth of greater than 100 million single-end 125 bp reads on an Illumina GAIIx sequencer, and aligned to the mouse genome (mm9) using TopHat with Bowtie [23]. To confirm that the majority of aligned sequence data came from spliced polyadenylated mRNA, we compared the ratio of intronic reads to exonic reads. For each intron in the 9,162 genes expressed at more than a mean of 20 reads per exonic nucleotide, we calculated the number of reads in the last 25 bp of each intron and divided it by the number of reads in the adjacent first 25 bp of the exon, to yield the ratio of reads across the 3′ splice site (3′SS ratio [7,9]) (Figure 1). This showed that almost all mRNAs are completely spliced with a mode of the intron retention less than 0.01 and a median of 0.019.

**Figure 2:**
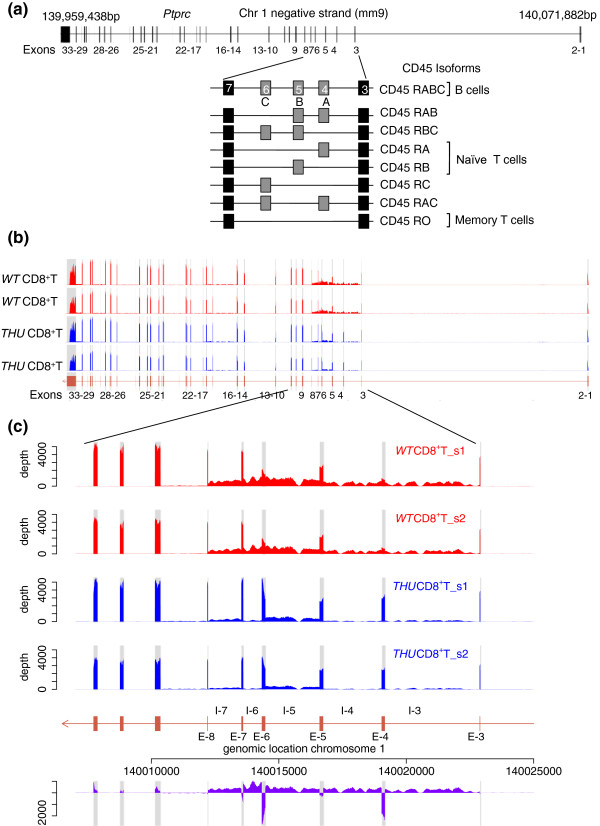
**Increased intron retention in wild-type T cells correlates with exon-skipping events in *Ptprc*. (a)** Exons 4, 5 and 6 of the *Ptprc* (CD45) gene are alternatively spliced in T cells and may be combined to produce eight distinct CD45 isoforms. The longest isoform, CD45RABC, is primarily expressed in B cells whereas T cells express different CD45 isoforms through their development and activation. **(b)** RNA-seq data along the length of the *Ptprc* gene show the introns flanking alternative exons 4, 5 and 6 are covered by a greater depth of sequence reads and indicates incomplete splicing of these introns in both wild-type (*WT*; in red) and *thunder* (*THU*; in blue) CD8^+^ T cells. **(c)** Read depth over retained introns is consistent within biological replicate samples, but is different between wild-type (red) and *thunder* (blue) samples, as shown in the purple trace, which plots the wild-type minus *thunder* per nucleotide read depth through the alternatively spliced region of the *Ptprc* gene. Introns and exons are labeled as I- and E-, respectively, followed by their corresponding number. *Thunder* mutants with a hypomorphic *Hnrpll* gene show fewer reads aligned to introns 3, 4, 5, 6 and 7.

The *Ptprc* gene contains 33 exons spanning 112 kb on chromosome 1 (Figure 2a), and is highly expressed in T cells with a median of >3,300 reads per exonic nucleotide in each of our CD8 T-cell RNA-seq datasets. The majority of *Ptprc* introns were spliced out of 99% of the sequenced RNA, as illustrated by the large difference in read coverage over constitutive exons 9 to 29 compared to the intervening introns (Figure 2b) and by a median 3′SS ratio of 0.01 for *Ptprc* introns as a whole. In wild-type CD8^+^ cells (CD45-RB^high^, RA^low^ and RB^low^) there was strong silencing of exons 4 and 6, which contain multiple ARS, whereas the more abundant exon 5, encoding the CD45-RB isoform, was better represented in sequence reads. The hnRNPLL-dependence of this differential splicing is readily apparent from Figure 2c - the two wild-type T cell samples had a mean of 777 and 1,639 reads per nucleotide in exons 4 and 6, respectively, compared with a mean of 3,735 for all *Ptprc* exons, whereas in the *Hnrpll*^*thu*^ T cells exon 4 and 6 were covered by a mean of 2,747 and 3,766, respectively, compared to a mean of 3,766 for all *Ptprc* exons.

Surprisingly, an abundance of reads was also observed in wild-type T cells selectively over *Ptprc* introns 3, 4, 5, 6 and 7 compared with the other introns in the gene. The median read depths over introns 3, 4, 5, 6 and 7 were 310, 366, 746, 759 and 552 reads, respectively, and were significantly higher (*P* = 1.6 × 10^-4^, Mann-Whitney, W = 200) than the mean depths over all other introns (median read depth of 16). Mutant *Hnrpll*^*thu*^ cells also showed significantly increased reads in the same introns, though at a universally lower level than wild-type cells. Hence, wild-type CD8^+^ T cells display a greater depth of intronic reads and inversely fewer exonic reads relative to mutant cells through the region of the *Ptprc* transcript known to undergo alternative splicing. The median read depth over these retained introns in wild-type cells is between 8 to 20% as abundant as sequence reads over all *Ptprc* exons. This indicates that, in wild-type CD8^+^ T cells, approximately 8 to 20% of the sequenced *Ptprc* mRNA had not yet removed either exon 4 and exon 6 nor the introns that precede and follow these exons, yet 99% of the sequenced *Ptprc* mRNA had removed constitutively spliced introns. It is important to note that deep sequencing of RNA provides a unique opportunity to measure mammalian intron retention in a relatively unbiased manner, in contrast to PCR-based methods that are biased against detecting the very large intronic inserts.

**Figure 3:**
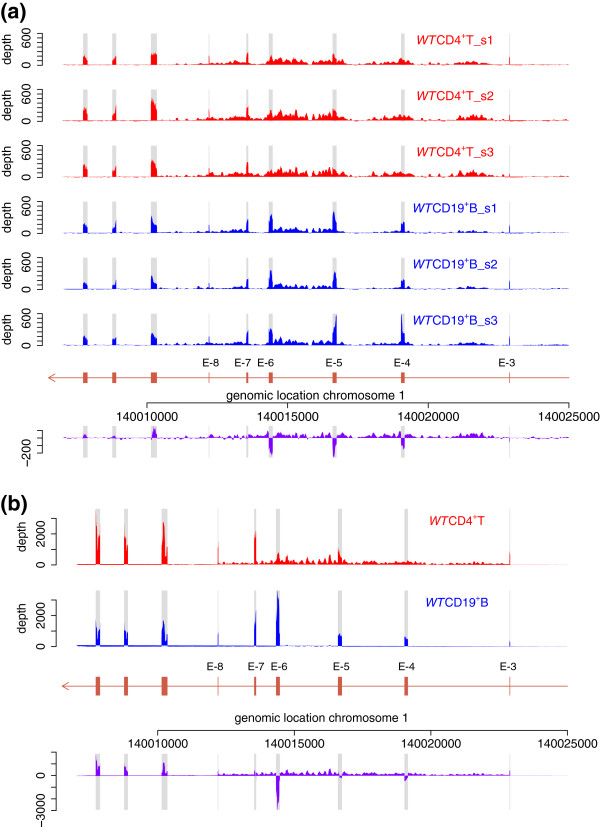
***Ptprc *intron retention correlates with hnRNPLL expression in wild-type T cells and low expression in B cells. (a)** CD19^+^ B cells (blue traces), which have naturally low expression of *Hrnpll*, display decreased intron retention compared with CD4^+^ T cells. The schematic diagram shows RNA-seq data prepared from three independent samples of wild-type lymphocytes enriched by magnetic beads for CD4^+^ T cells or CD19^+^ B cells. The difference in intron and exon read depths between T and B cells is shown in purple. **(b)** The pattern of decreased intron retention is recapitulated in an independent RNA-seq dataset from the ImmGen project [24] for wild-type T and B cells purified by fluorescence-activated cell sorting. T cells (red trace) show increased read depths over introns flanking the alternative exons of *Ptprc*, compared with B cells (blue trace). The difference in read depth over introns and exons is indicated by the purple trace.

As an independent test of the effect of diminished hnRNPLL activity on lymphocyte mRNA splicing and intron retention, we compared RNA-seq data from magnetic-bead enriched CD4^+^ T cells (*Hnrpll-*high) and CD19^+^ B-cells (*Hnrpll*-low) (Figure 3a). As expected for bead-based enrichment methods, the B-cell-enriched samples still contained an estimated 7% T cells based on mean expression of a set of T-cell-specific mRNAs, assuming exclusive T-cell expression of these genes. Conversely, using the same measure, the T-cell-enriched samples retained an estimated 1% B cells. In CD19^+^ B cells, the median sequence read depth over *Ptprc* exons 4, 5 and 6 was 344, which is greater than the median read depth of 165 over all *Ptprc* exons, demonstrating strong inclusion of these three exons as expected, since B cells express very little hnRNPLL. In CD4^+^ T cells, which do express hnRNPLL, the read depth over exons 4 and 6 was lower (median 130) than the median depth over *Ptprc* exons as a whole (192), confirming silencing of these exons. A reciprocal pattern was observed for the introns flanking exons 4 to 6, where there was a higher depth of reads in T cells (median of 54) and a lower depth of reads in B cells (median of 23). Hence, B cells with naturally low *Hnrpll* expression splice out introns 4 to 6 more efficiently than T cells that express *Hnrpll*.

As further evidence of differential intron retention between T and B cells, we analyzed RNA-seq data generated by the ImmGen project [24] from polyadenylated mRNA isolated from highly purified CD4^+^ T cells and CD19^+^ B cells prepared using fluorescence-activated cell sorting (Figure 3b) [25]. *Ptprc* introns 4 to 6 exhibited numerous reads in T cells, with a median of 144 compared to 1,655 for all *Ptprc* exons. By contrast, very few intron 4 to 6 reads were obtained from B-cell mRNA, with a read depth of 5 compared to a median of 1,071 for all *Ptprc* exons in the same sample. This indicates that introns 4 through 6 are efficiently spliced out of 99.5% of *Ptprc* mRNA in B cells that have little hnRNPLL, but are retained in 9% of the corresponding mRNA from CD4 T cells.

### Retained introns are frequent and correlate with alternatively spliced exons

**Figure 4:**
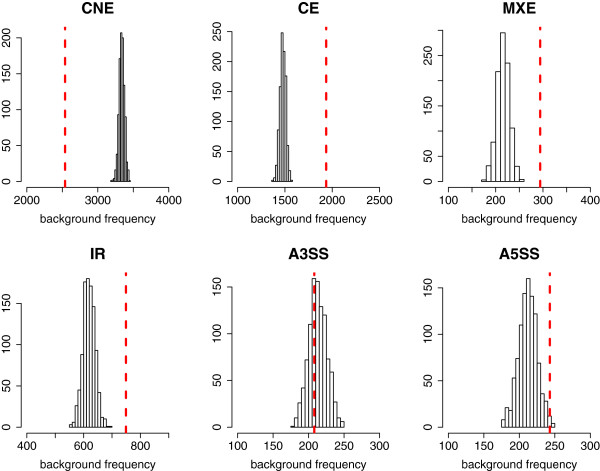
**Intron retention ratio is correlated with cassette exon, mutually exclusive exon and intron retention splicing events.** Frequency of matched alternative splicing event types in 1,000 random re-sampling exons representing the background distribution is compared with the observed number of each splicing event (highlighted in red dotted line) for the exons with increased intron retention (3′SS IR ≥0.01; CNE, constitutive exon; CE, cassette exon; MXE, mutually exclusive exon; IR, intron retention; A3SS, alternative 3′ splice site; A5SS, alternative 5′ splice site; Table 1). CE, MXE and IR types are significantly enriched, whereas CNE is significantly reduced compared with background (at false discovery rate *P*-value = 0.001). A3SS and A5SS are not found to be enriched.

Given the findings above, we asked how many other mRNA species show a retained intron in normal mouse T cells. The 3′SS ratio data shown in Figure 1 suggest that up to 13% of all introns in polyadenylated mRNA remain unspliced in more than 10% of the corresponding mRNAs (3′SS ≥0.1). A smaller subset of introns (0.94% of all introns) remain unspliced in more than 50% of the mRNAs that contain them. We therefore asked if exons observed with a retained flanking intron commonly undergo alternative splicing, by generating a list of exons that had a 3′SS ratio ≥0.1 in wild-type CD8 T cells from OT1 mice. In total, 6,797 exons had a 3′SS ratio greater than 0.1, derived from 3,248 genes (Figure 4, Table 1). The median intron retention (IR) ratio in detectable genes was 0.019 in wild-type T cells. When compared with annotated alternative exons in the Ensembl mouse gene set [26] (NCBIM37, Ensembl release 67), this list showed a highly significant, non-random overlap with the alternative splicing categories of cassette exons, mutually exclusive exons and intron retention (according to the nomenclature of [27]) whereas constitutive exons were found significantly less than expected (permutation *P*-value = 0.001; Figure 4, Table 1). The alternatively spliced *Ptprc* exons 4 and 6 that are skipped in *Hnrpll* wild-type T cells had a 3′SS ratio of 0.7 and 0.5, respectively, placing them within the top 8% of all exons ranked by largest 3′SS ratio. Hence, 3′SS ratios as a simple measure of intron retention can be used to enrich for mammalian exons that undergo physiological alternative splicing.

**Table 1:** Intron retention ratio is correlated with cassette exon, mutually exclusive exon and intron retention splicing events

**Alternative splicing event type**	**Number of annotated events in ENSEMBL for all exons (n = 38,911)**	**Number of matched events for exons with 3′SS IR ≥0.10 (n = 6,658)**	**Mean number of matched events in background (1,000 × re-sampling 6,658 exons from 38,911)**	**Permutation *P*-value**
CNE	19,513	2,538	3,338	0.001
CE	8,641	1,936	1,479	0.001
MXE	1,265	294	216	0.001
IR	3,614	749	620	0.001
A3SS	1,249	208	213	0.636
A5SS	1,242	243	213	0.009

Number of alternative splicing event types mapped within 1) all exons in expressed genes and 2) exons having at least 10% of intron retention in their 3′SS, and the mean number of the event types within randomly sampled exon sets of the same size. CNE, constitutive exon; CE, cassette exon; MXE, mutually exclusive exon; IR, intron retention; A3SS, alternative 3′ splice site; A5SS, alternative 5′ splice site.

### Detection of differentially retained introns in *Hnrpll*^*thu*^ mutants

The data presented above imply that binding of hnRNPLL to exons in pre-mRNAs delays the excision of flanking introns from polyadenylated mRNA. We therefore sought other hnRNPLL-regulated genes using algorithms to detect differential intron retention or exon exclusion between wild-type and *Hnrpll*^*thu*^ CD8^+^ T cells. Using the DEXSeq method [28] we compared the relative intron read counts within each annotated mouse gene between wild-type and *Hnrpll*^*thu*^ CD8^+^ T cells from OT1 mice. This identified 138 introns from 114 genes with significantly different numbers of reads between wild type and mutant, including intron 6 from the *Ptprc* gene, which ranked second most differentially retained according to *P*-value (*Ptprc* introns 3, 4, 5 and 7 were also within the top six most significantly retained introns; Additional file 1). Intron 1 of the *Ctse* gene was more highly retained than all *Ptprc* introns, and introns from the *Ddb2*, *Senp2*, *Trim30a* and *Atp2c1* genes comprised the remainder of the top 10 highest ranked introns by DEXSeq. Visual inspection of reads for these and other highly ranked genes using the Integrative Genomics Viewer (IGV) [29] showed clear cases of differential intron retention between wild-type and *Hnrpll*^*thu*^ T cells, although these were generally less striking than that observed in *Ptprc*, most likely due to these mRNAs being less abundant.

To identify differential exon exclusion we used DEXSeq to compare the relative read depths for each exon within annotated mouse genes between wild-type and *Hnrpll*^*thu*^ T cells, and ranked exons with significant differences according to *P*-value (Additional file 2). *Ptprc* exons 4 and 6 were ranked first and second, respectively. Of the 138 introns with DEXSeq evidence of differential intron retention above, 18 of these had at least one flanking exon with evidence of differential exon exclusion. These intron-exon pairs were from the *Ptprc*, *Degs1*, *Ms4a6c*, *Cdc42*, *Il7r*, *Ctse*, *Gimap8*, *Slfn1*, *Rpl29*, *Mrpl3* and *Ighg2c* genes.

As a third method to identify hnRNPLL-regulated splicing events, we surveyed differential splice junction usage. We used DEXSeq to compare the reads aligned to all splice junctions between wild-type and *Hnrpll*^*thu*^ T cells, which found 588 significant junctions out of 230,416 in total (at a false discovery rate of 0.1), three-quarters of which show increased usage in T cells from *thunder* mice, indicating greater splicing diversity is associated with mutant hnRNPLL (Additional file 3). Among 57 junctions that were equally highest-ranked with an adjusted *P*-value of 0, splice junctions that connect alternative exons in the *Ptprc* gene were found with a reciprocal pattern of exon-joining versus exon-skipping. Junctions joining exon 8 and exon 3, exon 7 and exon 3, exon 7 and exon 5, and exon 5 and exon 3 had higher relative sequence coverage in wild-type T cells, whereas junctions joining exon 6 and exon 5, exon 5 and exon 4, and exon 4 and exon 3 had higher coverage in *Hnrpll*^*thu*^ T cells. Of the top ten genes ranked by evidence of intron retention above, five of these also contained significant differentially altered splice junction usage flanking these introns (*Ptprc*, *Ctse*, *Trim30a*, *Rab3gap2* and *Slc12a7*).

Figure S1 in Additional file 4 shows a Venn diagram of the overlap of gene identifiers with significant differential intron retention, exon inclusion or alternative splice junction ratios. While a total of 9 genes (*Ptprc*, *Ctse*, *Ighg2c*, *Wdr82*, *Ms4a6c*, *Gnai2*, *Pik3r4*, *Degs1*, *Rrp9*) have significant differential intron retention, exon inclusion and altered splice junction usage (and another 51 with just two of these features), they are a minority compared with the total count of genes with one of these features. Hence, either methodology issues and noise in the RNA-seq data may hamper the identification of alternative splicing events associated with intron retention, or intron retention may not always be associated with alternative splicing. It seems plausible that intron retention induced by hnRNPLL binding may delay splicing of certain introns, but may not ultimately alter the splicing fate of the pre-mRNA molecule.

### hnRNPLL-dependent splicing in mouse T cells

**Figure 5:**
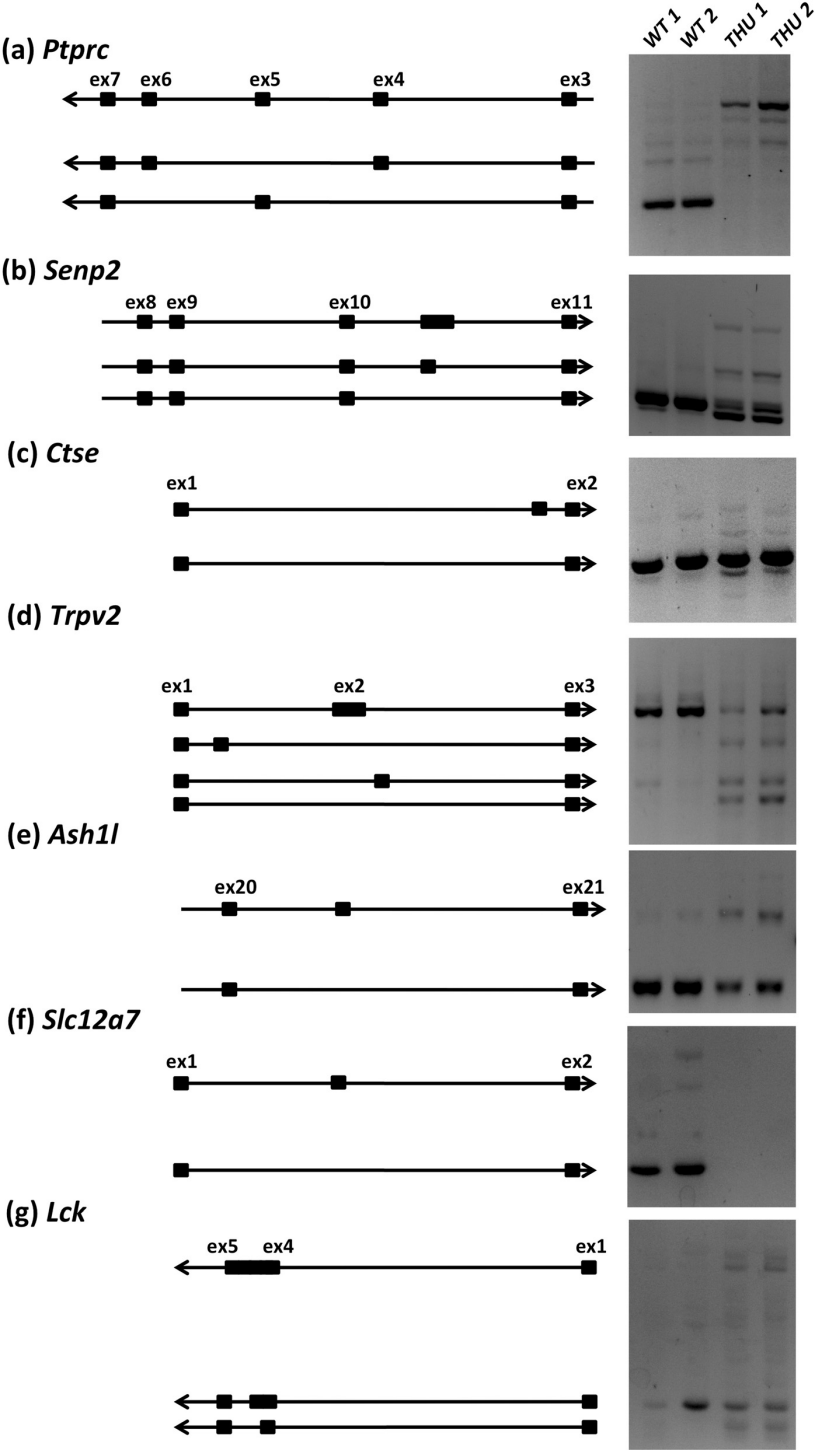
**Altered mRNA splicing between wild-type and mutant *Hnrpll *CD8**^**+ **^**T cells validated with RT-PCR.** A selection of ten genes that ranked highly in one or more tests for differential alternative splicing in *Hnrpll*^*thu*^ T cells were validated using RT-PCR. Six genes showed differential PCR bands between samples and are show alongside an ideogram of the inferred sequence included in each product, determined from band sizes and the expected included sequence indicated by RNA-seq junction read information. **(a)***Ptprc* gene, oligonucleotide PCR primers located in exons 2 and 7, amplifying across the regions of alternative exons 4, 5 and 6. **(b)***Senp2* gene, primers located in exons 8 and 11, amplifying the intron between exons 10 and 11 containing a variably included unannotated or cryptic exon. The sequence of each band in the accompanying ideogram was confirmed by Sanger sequencing. **(c)***Ctse* gene, primers located in exon 1 and 2, amplifying unannotated exon in intron 1. **(d)***Trpv2* gene, primers located in exons 1 and 3 to amplify unannotated exons in introns 1 and 2. **(e)***Ash1l* gene, primers located in exons 20 and 21, spanning variably included unannotated exon in intron 20. **(f)***Slc12a7* gene, primers located in an alternative first exon, such that preference to the other first exon there would be no product. **(g)***Lck* gene, primers were designed with a forward primer spanning across exons 1 and 4 and a reverse primer in exon 5, amplifying products of exon 1 and 4 joining (skipping exons 2 and 3) with variable length of exon 4. Oligonucleotide primer locations are displayed against read depth and gene intron/structure in Figure S2 in Additional file 4. WT, wild type.

The preceding analysis was validated in the sense that it identified known hnRNPLL-dependent splicing events in *Ptprc*, so we used reverse transcriptase polymerase chain reaction (RT-PCR) to validate highly ranked splicing events in other genes. Ten candidate alternative exons from distinct genes were selected, choosing those genes that ranked highly in one or more of the tests described in the previous section, and showed a clear differential pattern of read distribution between cell types when inspected visually using the IGV [29]. Oligonucleotides complementary to flanking exons were used to amplify PCR products from oligo-dT-primed cDNA prepared from *Hnrpll*^*wt*^ and *Hnrpll*^*thu*^ mRNA, and the products analyzed by gel electrophoresis (Figure 5). In addition to *Ptprc* as a positive control, six of the ten candidate genes, *Senp2*, *Ctse*, *Trpv2*, *Ash1l*, *Slc12a7*, *Lck*, displayed differential band sizes or multiple bands that differed between T cells with normal or mutant hnRNPLL (Figure 5). Interestingly, these genes all showed evidence for differential exon usage, but none showed bands attributable to retention of a long intron. This likely can be attributed to PCR bias towards shorter products and long intron-containing transcripts not being amplified, which is a bias not shared with RNA-seq. Alternative splicing events found in only three of these genes (*Ptprc*, *Trpv2*, *Slc12a7*) are currently identified by mouse expressed sequence tags (ESTs) from GenBank [30], indicating the need for more comprehensive annotation of alternative splicing by RNA-seq studies.

Browsing the RNA-seq reads data for these genes with IGV indicated that, like *Ptprc*, three genes (*Senp2*, *Ctse*, *Slc12a7*) appeared to have greater intron retention in wild-type cells than in *Hnrpll*^*thu*^ T cells, whilst the converse was true for two other genes (*Trpv2*, *Ash1l*) and indeterminate for one (*Lck*) (Figure S2 in Additional file 4). High intron retention and exon skipping occurred in wild-type T cells for *Senp2* and *Ctse*; for *Ash1l* and *Slc12a7*, however, high intron retention in *Hnrpll*^*wt*^ T cells was associated with exon inclusion. This indicates that either hnRNPLL binding can have both a splicing repressor and enhancer role in wild-type cells, or that potentially some of these genes are indirectly regulated by hnRNPLL or are regulated in concert with another RBP. For all validated candidate genes, the observed alternative splice junction usage from RNA-seq reads supports the alternative products observed with PCR.

For three candidate hnRNPLL targets (*Senp2*, *Ctse* and *Ash1l*) the alternative junctions detected by RNA-seq were outside the significance threshold applied in the DEXSeq analysis (Figure S2 in Additional file 4). Notable from the RNA-seq data for these genes was the enrichment of cryptic junction reads. Generally, cryptic splice sites are rare in the RNA-seq data, but four genes (*Senp2*, *Ctse*, *Trpv2*, *Slc12a7*) had a diverse number of cryptic splice junctions in their retained introns and flanking the identified alternative exons. Another feature of these differentially spliced genes was minor unannotated alternative exons. *Senp2*, *Ash1l* and perhaps *Slc12a7* and *Trpv2* have differentially included alternative exons that lie within retained introns. Within *Senp2*, a differentially included, unannotated, alternative exon appears in *Hnrpll*^*thu*^ cells, which is only present at trace levels in wild type. Further investigation of this cryptic exon in *Senp2* by sequencing PCR bands showed that it was included in variable lengths with alternative 3′ splice sites (Figure S3 in Additional file 4). Inclusion of this cryptic exon introduced an in-frame stop codon that we estimated caused 23% of the protein to be truncated in *Hnrpll*^*thu*^ cells (calculated as the percentage of cryptic junctions compared with canonical junctions).

**Figure 6:**
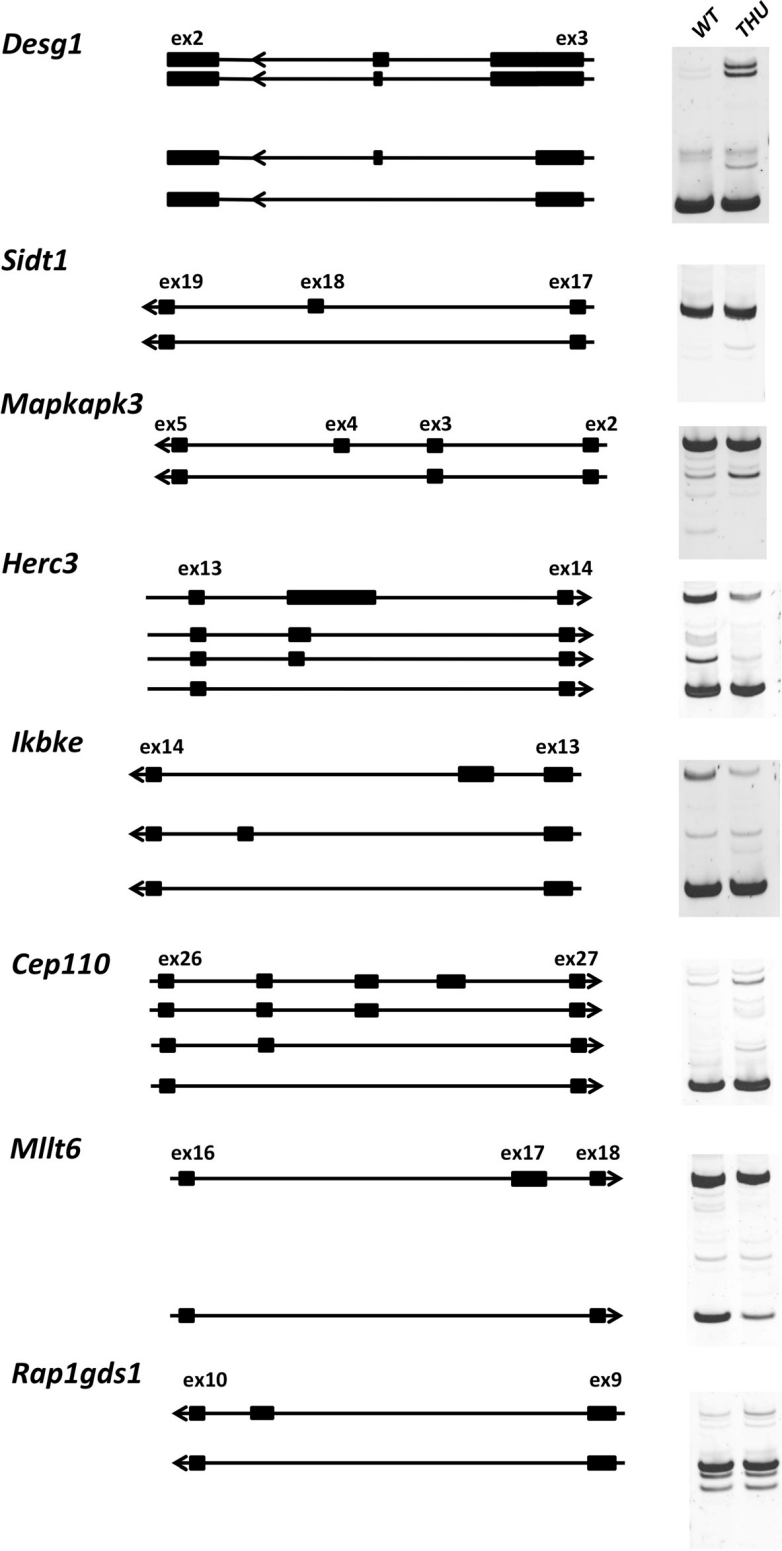
**RT-PCR validation of differential mRNA splicing of genes predicted by the Boolean scoring method.** Genes with RNA-seq features associated with differential exon inclusion and intron retention were scored and ranked alongside a set of seven genes that we validated by RT-PCR to be differentially spliced. Fifteen genes that ranked by score among these validated cases were selected for further RT-PCR, of which eight also showed differential banding. These products have been separated by eletrophoresis on acrylamide gels. Alongside each gel picture for each gene is an ideogram of the inferred sequence of each product, estimated by band size and the expected products predicted by junction read abundance from RNA-seq data. A single replicate from each genotype only was appraised, and these correspond to wild-type (WT) replicate 2 and *thunder* replicate 2 in Figure 5.

By combining each of the features of hnRNPLL-dependent alternative splicing events observed above, we developed a simple scoring scheme based on the sum of Boolean values for each feature (Table 2). These features were intron retention, exon exclusion and splice-junction usage (determined by DEXSeq) along with the presence of cryptic junctions and high 3′- or 5′SS ratios. We scored all introns by these features and ranked them accordingly. *Ptprc* and four other PCR-validated genes (*Senp2*, *Ctse*, *Ash1l*, *Slc12a7*) ranked within the top 25, while *Trpv2* and *Lck* ranked 63 and 164, respectively. A further 136 genes had introns with scores that ranked them among the validated genes and were likely candidates for hnRNPLL-dependent splicing. We selected 15 of these candidate genes after visual inspection of the RNA-seq read data with IGV and conducted further RT-PCR validation of alternative splicing. The results identified 8 of these 15 genes (*Degs1*, *Sidt1*, *Mapkapk3*, *Herc3*, *Ikbke*, *Cep110*, *Mllt6*, *Rap1gds1*) with differential band sizes between *hnrpll*^wt^ and *hnrpll*^thu^ T cells (Figure 6). Table 2 shows the scoring matrix of Boolean values of these high ranking genes confirmed by RT-PCR. Across these genes and those identified earlier, gene set enrichment analysis did not indicate significant shared Gene Ontology terms, common pathways or other functional annotations.

**Table 2:** All candidate hnRNPLL-regulated splicing events prioritized for RT-PCR confirmation

**Gene**	**Intron location**	**Feature scores**						**Score**	**Rank**
		**3′SS ratio**	**5′SS ratio**	**Differential exon**	**Differential intron**	**Differential junction**	**Cryptic junctions**		
*Ptprc*	1:140013603-140014330	1	1	1	1	1	1	6	1
*Degs1*	1:184207025-184208627	1	1	1	1	1	1	6	2
*Ptprc*	1:140016744-140019032	1	1	1	1	1	0	5	3
*Ptprc*	1:140012220-140013530	1	0	1	1	1	1	5	4
*Ptprc*	1:140019162-140022879	1	0	1	1	1	0	4	6
*Slc12a7*	13:73870786-73901144	1	0	0	1	1	1	4	7
*Slc12a7*	13:73901339-73921966	0	1	0	1	1	1	4	8
*Ptprc*	1:140014472-140016596	1	1	1	1	1	0	5	11
*Rab3gap2*	1:187087739-187090779	1	0	0	1	1	1	4	13
*Il7r*	15:9440015-9440865	0	0	1	1	0	1	3	15
*Ctse*	1:133535135-133558452	0	0	1	1	0	1	3	16
*Sidt1*	16:44258155-44259545	0	0	0	1	1	1	3	17
*Il7r*	15:9438538-9439920	0	0	1	1	0	1	3	20
*Il7r*	15:9438114-9438461	0	0	1	1	0	1	3	21
*Ash1l*	3:88871164-88872908	0	1	0	1	0	1	3	23
*Senp2*	16:22032238-22036542	1	1	0	1	0	1	4	24
*Rnf167*	11:70463837-70464265	0	1	0	0	1	1	3	26
*D14Abb1e*	14:28263236-28272964	0	0	0	0	1	1	2	33
*Sept7*	9:25104073-25105396	0	0	0	0	1	1	2	37
*Mapkapk3*	9:107166096-107182976	0	1	0	1	0	1	3	44
*Trim68*	7:109828211-109828650	1	1	0	0	1	1	4	48
*Sigirr*	7:148281217-148286377	1	0	0	0	1	1	3	50
*Trpv2*	11:62388103-62389048	1	0	0	0	1	1	3	62
*Herc3*	6:58824395-58826482	1	0	0	1	0	1	3	66
*Ighg2c*	12:114524254-114525607	0	1	1	1	0	0	3	71
*Pik3r4*	9:105580651-105584514	0	0	0	1	0	1	2	80
*Ikbke*	1:133162866-133166377	0	0	0	1	0	1	2	83
*Cep110*	2:35013309-35015767	0	0	0	0	1	1	2	84
*Fcrl1*	3:87185307-87185682	0	0	0	0	1	1	2	87
*Mllt6*	11:97538551-97539627	0	0	0	1	0	1	2	104
*Rap1gds1*	3:138619284-138620373	0	0	0	0	1	1	2	131
*Lck*	4:129234700-129234869	0	0	0	0	1	1	2	163

Number of alternative splicing event types mapped within 1) all exons in expressed genes and 2) exons having at least 10% of intron retention in their 3′SS, and the mean number of the event types within randomly sampled exon sets of the same size.

### Normally spliced *Senp2* cDNA restores *Hnrpll*^*thu*^ T-cell survival

**Figure 7:**
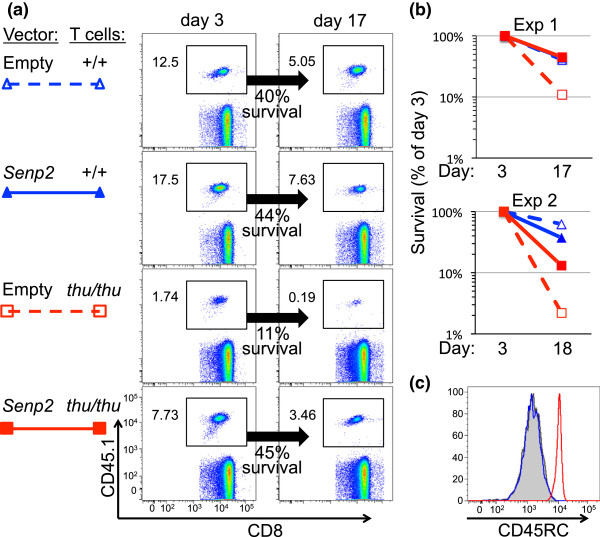
**Expression of normally spliced *Senp2 *cDNA partially restores survival of *Hnrpll***^***thu***^**T cells.** CD8 T cells from *Hnrpll*^*+/+*^ or *Hnrpll*^*thu/thu*^ OT-1 TCR-transgenic B6.SJL-CD45.1/CD45.2 mice were activated and spinoculated with defective retroviral particles containing either empty retroviral vector or the same vector bearing full length, normally spliced *Senp2* cDNA, and injected into the circulation of normal C57BL/6 mice. **(a)** Blood from each recipient mouse was analyzed by flow cytometry 3 and 17 days later, enumerating the frequency of donor CD45.1+ T cells among CD8 cells at each timepoint and the percentage that survived between days 3 and 17. **(b)** Data from two independent experiments analyzed as in (a). **(c)** Staining of the donor-derived T cells for CD45RC: black and blue histograms, +/+ cells with empty or *Senp2* vectors, respectively; red histogram, *thu/thu* cells with *Senp2* vector.

The lifespan of *Hrnpll*^thu^ homozygous T cells is greatly shortened, resulting in decreased numbers of naïve T cells in the circulation [16] due to effects on hnRNPLL targets other than *Ptprc*[22]. We focused on *Senp2* as an additional candidate, since it was highly ranked and validated with strong evidence of intron inclusion and cryptic splicing that would decrease the pool of normal Senp2 protein in the analysis of *Hrnpll*^thu^ homozygous OT-1 T cells above, and because it is an essential regulator of protein sumoylation with the capacity to affect many aspects of cell survival in non-lymphoid cells [31,32]. OT-1 CD8 T cells with wild-type or homozygous mutant *Hnrpll* were activated briefly in culture, transduced with a retroviral vector encoding normally spliced *Senp2* cDNA or an empty vector control, and re-implanted into normal C57BL/6 mice. Individual transferred CD8 T cells were enumerated in blood samples collected from the recipients on day 3 and again on day 17, using flow cytometric staining for a donor-specific CD45.1 marker (Figure 7a). Wild-type T cells transduced with *Senp2* or empty vector were frequent in the circulation on day 3, and persisted at 40% of this frequency on day 17. *Hnrpll* mutant T cells transduced with empty vector were less frequent on day 3, and had declined to 11% of this frequency by day 17, consistent with previous evidence that these T cells have a very short *in vivo* lifespan [16]. By contrast, mutant T cells transduced with *Senp2* vector were present at higher frequency on day 3 and persisted at 45% of this frequency by day 17. Improved survival of *Hnrpll* mutant T cells transduced with Senp2 compared with control vector, but not of *Hnrpll* wild-type T cells transduced with the same vector combination, was observed in two independent experiments (Figure 7b). Flow cytometric staining of the persisting *Hnrpll* mutant T cells showed that they nevertheless continued to be unable to silence *Ptprc* exon 6, displaying 10 times higher CD45RC on their cell surface compared with the transferred wild-type T-cell controls (Figure 7c). This result, together with isolation of an ENU-induced exon 10-11 splicing mutation in *Senp2* that also results in shortened T-cell survival (Yan Mei, Anselm Enders, Edward M Bertram and Christopher C Goodnow, unpublished observations), provide evidence that *Senp2* is an important functional target for *Hnrpll*-dependent T-cell survival.

## Discussion

Our study develops a strategy for identifying regulated mRNA splicing events with RNA-seq of total polyA + RNA, using one of the best defined mammalian models of developmentally regulated alternative splicing in the *Ptprc* gene and a mutation that cripples hnRNPLL, a key RBP regulating this event. We have shown that introns flanking the excluded exons in this gene are retained in oligo-dT primed cDNA from T cells that express hnRNPLL, whereas these introns are efficiently spliced out of most of the mRNA in lymphocytes with defective or naturally low expression of hnRNPLL. Intron retention promoted by hnRNPLL appears to be a general feature of the transcriptome in T cells and we identified and validated a further 14 genes showing a similar pattern of intron retention and alternative splicing. Functional evidence for the importance of one of the highest-ranked additional targets, *Senp2*, was obtained by retroviral cDNA expression restoring survival of *Hnrpll*-mutant T cells. The computational methodology we have applied to identify hnRNPLL-dependent splicing events in comparison between wild type and mutant cells is readily generalizable to identify the program of other regulatory RBPs that dictate patterns of alternative splicing, using small samples of total RNA that are readily obtained from purified *ex vivo* subsets of primary mammalian cell types.

We have shown that partially spliced polyadenylated mRNAs, rather than being artifacts of RNA-seq data, serve as markers of alternatively spliced exons and that the retention of introns in these transcripts is a regulated process. Previous studies of nascent mRNA have employed technically demanding nuclear fractionation before RNA isolation [7,8,33]. Our results are built on an emerging literature that demonstrates incompletely spliced, polyadenylated mRNAs - including unspliced introns 3 to 7 in *Ptprc* mRNA from macrophages - are frequently retained on chromatin as well as being present in the nucleoplasm [8,10,33,34]. Incompletely spliced, polyadenylated mRNAs are readily identified in RNA-seq datasets generated from whole-cell mRNA preparations [10,35,36] and differential intron retention between different tissues and cell types has also been observed [7,13,36]. The cause of the differing intron excision in these studies is mostly unresolved and may stem from various factors, including intron length, strength of 5′ splice sites, RNA polymerase II pausing, and the binding of SR proteins to introns [33]. Our study addresses this question by establishing a strong correlation between intron retention in total polyA + RNA and the presence of variably spliced exons, and by experimentally demonstrating the action of a developmentally regulated, RNA-binding splicing silencer, hnRNPLL. Nuclear fractionation and sequencing of nascent, chromatin-associated transcripts and those from the nucleoplasm will provide a higher degree of sensitivity for detecting unspliced transcripts, but the high quantity of input material required is unfeasible for cells of limited abundance. The diversity of RBPs in mammalian cells allows strong cell-type-specific regulation of splicing, as we have found, and produces an imperative to compare purified cell populations, which will frequently mean that starting cells will be in limited supply.

Along with this simple means of generating data we have developed a scoring and ranking scheme to identify genes displaying properties associated with regulated intron retention and alternative splicing. No single feature of exon inclusion, intron retention or splice junction usage was solely sufficient to reliably identify a group of transcripts as undergoing regulated late splicing, but a simple integration of evidence from RNA-seq data could better rank genes. From a set of six genes with RNA-seq and RT-PCR evidence for differential splicing between wild-type and *thunder* Hnrpll mRNAs, we identified that cryptic junction reads are a frequent feature of introns flanking alternative exons. Machine learning approaches are often used to take information about a training set of genes to extrapolate further similar cases across a large dataset. We applied a naïve Bayesian classifier to use these six genes to identify further targets of hnRNPLL regulation. Perhaps due to the small number of training genes we found the classifier did not produce a list of genes with convincing evidence on visual inspection of the RNA-seq data in IGV. As more studies elucidate the features of alternatively spliced genes regulated by RBPs, a greater number of known, training genes may enable better use of machine learning algorithms.

Regulated retention of introns flanking alternative exons in the *Ptprc* gene indicates that splicing decisions are frequently delayed until after transcription and polyadenylation. One suggested role for post-transcriptional splicing is that it provides more (or later) opportunities for regulated alternative splicing [10]. The retained introns we have observed in the *Ptprc* gene do not appear to be ‘dead-end’ transcripts destined for degradation, but are likely exons on which a splicing decision is pending. Speculatively, delayed processing of variable exons in *Ptprc* may provide a pool of mRNA that can be rapidly modified in response to changing extracellular signals. This paradigm is perhaps best demonstrated by the unconventional cytoplasmic splicing of *Hac1/Xbp1* mRNA in response to endoplasmic reticulum stress [37], but has also been suggested for conventional alternative splicing [13]. While there is no direct evidence to support this possibility for *Ptprc*, TCR-induced dephosphorylation of PSF1 promotes *Ptprc* exon silencing [38]. Potentially, this pool of ‘delayed transcripts’ are awaiting a cellular decision on the splicing of alternative exons. In the absence of this decision introns surrounding the alternative exons remain unspliced, to allow the option of a splicing event that causes an exon to be skipped.

The differentially spliced genes between wild-type and *thunder* T cells elucidate the primary or secondary targets of hnRNPLL and identify a cellular program that is likely to be specifically regulated between naïve and activated/memory T cells. Both SR proteins and hnRNPs are encoded by large gene families and the diversity of these proteins allows highly tissue-specific splicing regulation by single RBPs [39]. Hence, we need not expect a large number of hnRNPLL-dependent targets. However, apart from these genes all being co-expressed in T cells, there are few annotations that obviously link them functionally as a larger program. Several pairwise functional associations are clear, such as the well characterized direct interaction of CD45 with LCK to dephosphorylate pTyr-505 and pTyr-394 regulatory tyrosines on LCK [40,41]. One hypothesis to explain this apparent lack of a broader program may be that the true functional targets of hnRNPLL are indeed a very small set, perhaps being just one, two or three genes. Functional associations between these genes will not be effectively captured through gene set enrichment analysis, as gene pairs or trios will be statistically very sensitive to missing or incomplete functional annotations. The differential exon usage in the *Ptprc* gene is the clearest signal seen in the RNA-seq data and the signal of the other genes is much less distinct, and decreases quickly to the limit of detection through our positive gene list. It could be that genes with weaker signals represent minor or promiscuous binding targets of hnRNPLL without strong functional effect. Of the stronger targets, namely those in our first list of six high ranking gene targets, it will be important to investigate the functional impact of the splicing regulation mediated by hnRNPLL. We provide evidence here that inefficient splicing of one of these targets, *Senp2*, contributes to the short lifespan of hnRNPLL-mutant T cells. *Senp2* encodes a sumo-specific protease and results in embryonic lethality when knocked out [31,32], but how it promotes T-cell longevity will await further study.

Detection of genes with differentially retained introns from RNA-seq data suggests a method for mapping the targets and extended regulatory program of hnRNPs and potentially some SR proteins. This approach identifies both direct and secondary targets of RNA binding proteins, and hence would be highly complementary to additional sources of information from RIP-seq, PAR-CLIP and other immunoprecipitation followed by sequencing approaches. Collections of mice with knockout and mutant hnRNPs are steadily growing and transcriptome data taken from appropriate cells could be extracted, sequenced and mined using a technically simple approach similar to that presented here. For example, hnRNPLL and hnRNPL have overlapping roles in regulating the splicing of *Ptprc* in T cells [42]. With existing mouse knockouts for hnRNPL, mapping of the functional targets of RBPs could be undertaken similarly to this study, in combination with genome-wide RNA-binding assays.

## Conclusions

Collectively, our data indicate that lymphocytes that express wild-type hnRNPLL at high levels (wild-type T cells) exhibit a delay in splicing of the *Ptprc* introns surrounding those exons (exons 4 and 6) with multiple hnRNPLL binding sequences, so that 8 to 20% of mRNA retains these introns after they have spliced out most other introns and become polyadenylated. This provides *in vivo* genetic evidence in mammalian cells that intron processing is delayed around alternatively spliced exons in nascent mRNA, whereas it often occurs co-transcriptionally and preceding polyadenylation for introns associated with constitutively spliced exons, consistent with emerging observations in other systems [7,8,33].

## Materials and methods

### RNA preparation

Single cell suspensions of mouse spleens were prepared by sieving and gentle pipetting followed by lysis of red blood cells. CD8^+^ T cells were isolated to >90% purity (as assessed by flow cytometry) using mouse CD8α microbeads (Miltenyi Biotec Australia Pty. Ltd. NSW, Australia) following the manufacturer’s instructions. Total RNA was then extracted using TRIzol (Invitrogen™ Life Technologies Australia Pty. Ltd. Victoria, Australia) and isopropanol precipitation. The purity and integrity of RNA was determined using the Agilent Bioanalyzer, which reported an RNA integrity number of >8.5 for all samples. CD19^+^ B cells and CD4^+^ T cells were purified from spleens of wild-type C57BL6 mice using microbeads conjugated with either anti-CD19 or anti-CD4 antibodies.

### Sequencing and mapping

Sequencing was done using an Illumina GAIIx with a single-end length of 125 bp for OT1 samples and 76 bp for non-OT1 samples. RNA-sequencing reads were aligned to the mouse reference genome mm9 (NCBIM37) using TopHat (v1.4.1) with Bowtie by using default parameters, which allows up to two mismatches [23]. Mean base coverage of an intron or exon was calculated as following: Number of reads mapped × Read length/Feature length. Gene expression was estimated from all reads mapping to all exons annotated to a gene. A minimum average coverage threshold of 20 bases was used to designate genes as expressed. The raw sequencing data have been uploaded to NCBI Short Read Archive under accession number SRP034881.

### Detection of differential splicing

Identification of differential inclusion of introns, exons and/or splice junctions was done using the DEXSeq package (version 1.8.0 on R version 3.0.2) [28], which normalizes count data for different coverage depths and estimates data dispersions, then tests for differential intron/exon/junction usage between wild type and mutant using a generalized linear model. An input dataset of non-overlapping exonic parts of the mm9 genome was created by the script dexseq_prepare_annotation.py provided in the package based on the Ensembl transcript annotation file Mus_musculus.NCBIM37.66.gtf, which was used to create a table of intronic parts for multi-exon genes. For more sensitive detection of differential reads within long introns, introns were further divided into 300 bp windows. The dexseq_count.py script, also part of the DEXSeq package, was then used to count the number of reads that fall within each exon or intron. Analysis of differential exon and intron inclusion were performed on both features simultaneously with DEXSeq (Additional file 5). As most introns contain few or no reads and pooling the data in this manner allowed better normalization. Dispersion estimation and statistical model calculation was performed using the TRT method provided with DEXSeq on exons and introns with a minimum of 10 counts over all samples. A raw splicing junction count dataset was generated using TopHat and was used in DEXSeq analysis to find differential splice junction usage for junctions of a minimum count of 2 over all samples. At a false discovery rate of 0.1, 558 out of 230,416 junctions were found to be significant.

### Calculation of intron retention ratio

Intron retention ratios across 5′ and 3′ splice sites were calculated as the sum of reads mapped to 25 bp upstream and downstream of the splice site, and calculated as the ratio of intronic reads to exonic reads, as previously described [7].

### Retained introns and annotated alternative splicing events

Exons associated with high 3′- or 5′SS read ratios were compared to alternative splicing events annotated to the Ensembl mouse gene set [26] (NCBIM37, Ensembl release 67). Annotated alternative splicing event types included constitutive exon (CNE), cassette exon (CE), mutually exclusive exons (MXE), intron retention (IR), alternative 3′ sites (A3SS), and alternative 5′ sites (A5SS) after the nomenclature described in [43]. A frequency of each splicing event type was calculated for exons that had a 3′SS ratio ≥0.01 in wild-type T cells from OT1 mice, then compared with 1,000 randomly permuted sets for each type of splicing event. *P*-values were calculated for a null hypothesis that the frequencies of splicing event types in exons having at least 10% of intron retention are not different to a background distribution, with an alternative hypothesis that the frequencies in exons of increased 3′SS IR is higher for event types CE, MXE, IR, A3SS, and A5SS and lower for the CNE type.

### Scoring scheme to rank differential intron retention events

Six measures related to differential intron retention were calculated: differential exon inclusion, differential splice junction usage, differential intron retention, distinct cryptic splice sites (per intron) and 3′- and 5′SS ratios. Differential exon, intron and splice junction read tests were calculated with DEXSeq as described above and features were scored ‘true’ if they were below a significance threshold equivalent to a false discovery rate of less than 0.1. Cryptic junctions were scored as ‘true’ if they had more than one unannotated junction supported by three or more reads in at least one sample. 3′- and 5′SS ratios were calculated as described above and these ratios compared between cells types. Where ratios differed by 0.1 or more, these were scored as ‘true’. Across all six feature types true values were summed and introns ranked according to total score.

### PCR validation of candidate genes

RNA samples from OT-1 CD8^+^ T lymphocytes from spleen of wild-type and *thunder* mice were prepared as described above then reverse transcribed to produce first-strand cDNAs (Marligen Biosciences, Ijamsville, MD, USA). cDNA products were quantified with a Nanodrop Spectrophotometer and used for PCR amplification of candidate genes using Taq DNA polymerase with a 55°C annealing temperature and 35 cycles, which were then electrophoresed in 2% agarose gel or 4% polyacrylamide gel, stained with GelRed or Syber Gold, respectively. Primer sequences used for validation of alternative splicing variants are shown in Table S4 in Additional file 4.

### OT-1 T cell transduction and transplantation

CD8 T cells were purified by magnetic bead depletion of other lymphocyte subsets from spleens of OT-1 TCR transgenic B6.SJL-CD45.1 congenic mice of *Hnrpll*^*+/+*^ or *Hnrpll*^*thu/thu*^ genotype. The T cells were activated by culture at 2 × 10^6^ cells/ml with plate-bound anti-CD3 and soluble anti-CD28 antibodies for 24 h, and then spinoculated with defective retroviral particles packaged in Phoenix cells containing either empty pMIG II vector or the same vector bearing full length normally spliced *Senp2* cDNA. The T cells were placed back in culture with anti-CD3 and anti-CD28 for 24 h, washed, and injected into the lateral tail vein of normal C57BL/6 mice. Each recipient mouse was bled 3 and 17 days later, blood cells stained for CD8, CD45.1 and CD45RC, and analyzed by flow cytometry.

## Abbreviations

ARS: activation response sequence; bp: base pair; hnRNP: heterogeneous ribonucleoprotein; IGV: Integrative Genomics Viewer; IR: intron retention; RBP: RNA-binding protein; RRM: RNA-recognition motif; RT-PCR: reverse transcriptase polymerase chain reaction; SR protein: Serine/arginine-rich protein; SS: splice site; TCR: T-cell receptor.

## Competing interests

The authors declare that they have no competing interests.

## Authors’ contributions

VC, AT, CCG and TDA designed the study. VC, YM, AT and AS performed the experiments. AM, SC, AE and EB produced and maintained mouse breeding lines. VC and TDA analyzed the data. VC, CCG and TDA wrote the paper. All authors read and approved the final manuscript.

## Authors’ information

Christopher C Goodnow and T Daniel Andrews are senior authorship.

## Supplementary Material

Additional file 1: Table S1 Table of differential intron usage found by DEXSeq. Click here for file

Additional file 2: Table S2 Table of differential exon usage found by DEXSeq. Click here for file

Additional file 3: Table S3 Table of differential junction usage found by DEXSeq. Click here for file

Additional file 4: Figure S1 A Venn diagram of genes with features (exon/intron/junction) found significant by DEXSeq. DEXSeq tests for finding differential exon, intron and junction inclusion resulted in a total of 127, 102, and 401 genes with significant features, respectively. Genes identified by all three tests are listed in group A, and by two tests are in group B, C and D. **Figure S2.** Representation of coverage depth and splice junction usage in genes with validated alternative splicing events by PCR. **Figure S3.** A cryptic exon inclusion in *Hnrpll*^*thunder*^ T cells is confirmed by sequencing PCR bands of *Senp2*. **Table S4.** A list of primer sequences used in validation of candidate genes with alternative splicing variants. Click here for file

Additional file 5 R log files for DEXSeq analysis of finding differential intron/exon and junction usage. Click here for file
