# Supplementary material for: The RNA-binding protein hnRNPLL induces a T cell alternative splicing program delineated by differential intron retention in polyadenylated RNA
Source: Genome Biol. 2014 Jan 29;15(1):R26. doi: 10.1186/gb-2014-15-1-r26 (PMC4053824; doi:10.1186/gb-2014-15-1-r26)
Supplement: Additional file 4: Figure S1 — A Venn diagram of genes with features (exon/intron/junction) found significant by DEXSeq. DEXSeq tests for finding differential exon, intron and junction inclusion resulted in a total of 127, 102, and 401 genes with significant features, respectively. Genes identified by all three tests are listed in group A, and by two tests are in group B, C and D. Figure S2. Representation of coverage depth and splice junction usage in genes with validated alternative splicing events by PCR. Figure S3. A cryptic exon inclusion in Hnrpllthunder T cells is confirmed by sequencing PCR bands of Senp2. Table S4. A list of primer sequences used in validation of candidate genes with alternative splicing variants. [file gb-2014-15-1-r26-S4.docx]

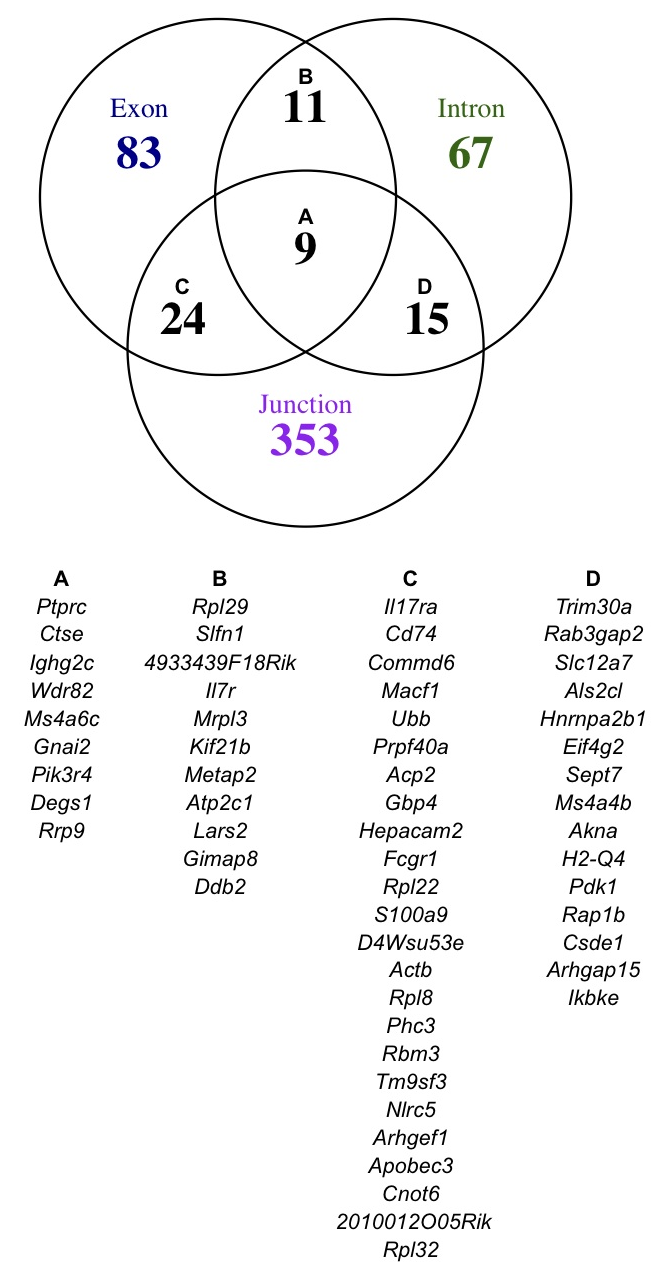


**Supp Figure 1**. **A Venn diagram of genes with features (exon/intron/junction) found significant by DEXSeq.** DEXSeq tests for finding differential exon, intron and junction inclusion resulted in a total of 127, 102, and 401 genes with significant features, respectively. Genes identified by all three tests are listed in group A, and by two tests are in group B, C and D.


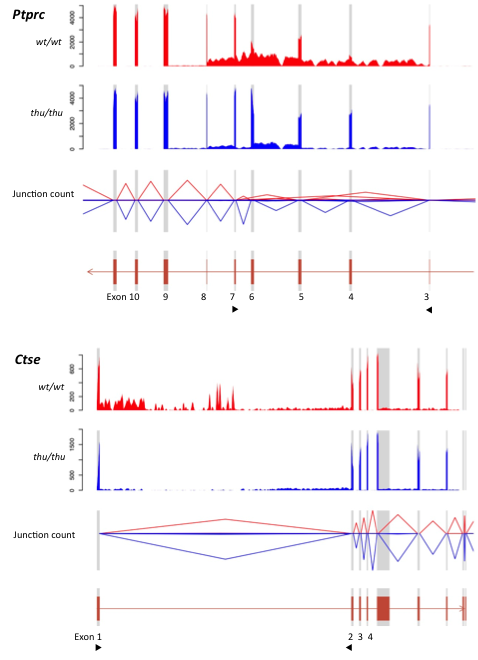

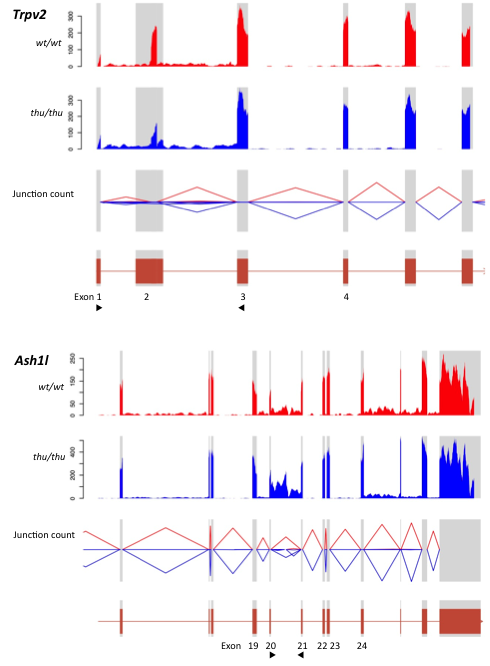

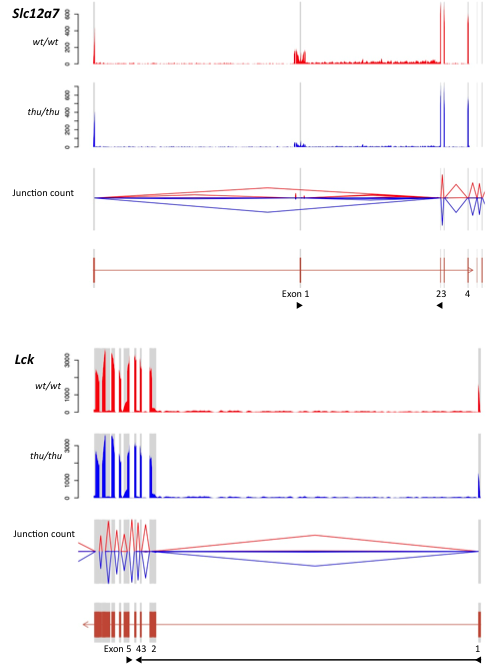


**Supp Figure 2.** **Representation of coverage depth and splice junction usage in genes with validated alternative splicing events by PCR.** For each gene, the top two panels show averaged coverage depth for wild-type and *thunder*, along with averaged splice junction counts where the peak heights are proportional to the total gene count (splice junctions are shown in apposite direction in red and blue for wild-type and *thunder*, respectively). Exons are numbered as in Figure 4 and the small triangles indicate locations of the left and right primer used in PCR.

**
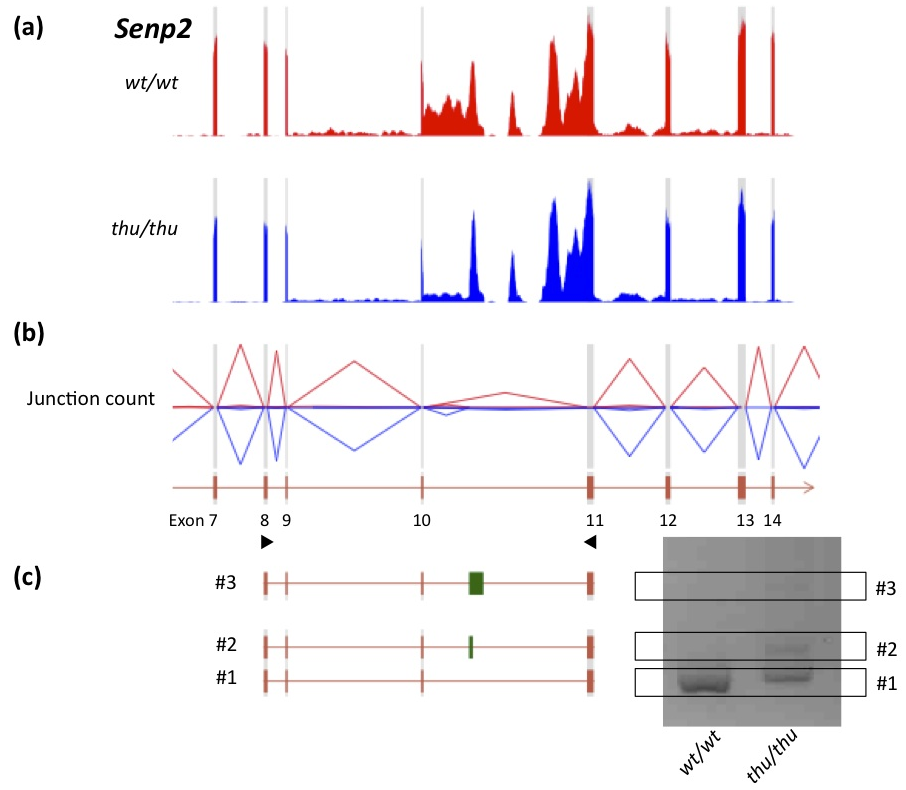
Supp Figure 3**. **A cryptic exon inclusion in *Hnrpll^thunder^* T cells is confirmed by sequencing PCR bands of *Senp2*.** **(a)** Averaged coverage depth for wild-type and *thunder*. **(b)** Averaged splice junction count. The peak heights are proportional to the total gene count (splice junctions are shown in apposite direction in red and blue for wild-type and thunder, respectively). Small black triangles represent left and right primer used in PCR, sitting on exon 8 and 11, respectively. **(c)** PCR followed by sequencing confirmed the presence of alternative products in the mutant – an unannotated cryptic exon inclusion (dark green), and less product of exon 10-11 (bands in box #1) in *thunder* compared to wild-type. The size of cryptic exon in the PCR bands shown in #2 and #3 is 95bp and 373bp, respectively. They share the same 5’ splice site with alternative 3’ splice sites, and located in between exon 10 and 11 of the *Senp2* gene. Immediately upstream of the cryptic exon shows differential intron coverage. These cryptic exons are skipped in wild-type T cells. The finding is also supported by increased splice junction of exon10-cryptic exons and decreased splice junction of exon10-11 in *thunder* (although they were below the significant threshold in DEXSeq junction analysis).

| **Gene name** | **Left primer** | **Right primer** |
| --- | --- | --- |
| *Ptprc* | GGCAAACACCTACACCCA | CCAGAGTGGATGGTGTAAGA |
| *Senp2* | GCGTCAGAACAACCCATTTT | CTGTCCTTCTCTTTGCTCGG |
| *Ctse* | CAGGCGTGGTTATCTCCATT | ACTCTGCTCCGATCTCCCTT |
| *Trpv2* | CAAACACAAGCAGAAGATGCT | GCACTGCCTTCTTCATCTCC |
| *Ash1l* | GTGTGAACACGGATGTGGAG | TGACGAAGCAGCAAGTCATC |
| *Slc12a7* | ATGCCCACGAACTTTACGG | CATGTTCTTCCCCTCGAAGT |
| *Lck* | AGGCTGGGCAGACAACCT | CCACGAAGTTGAAGGGAATG |
| *Il17ra* | CACAGTTCCCAAGCCAGTTG | GATGATCAGCACGATGACAGA |
| *Commd6* | GTCACGGGCCAGCTTATAGA | ATCTCGATGGACTTGCTGCT |
| *2010012O05Rik* | AGGCAGCTCGAAACATGGTA | GCTTCTTGCTGGTACTGAAGG |
| *Degs1* | TCTTGAAGGGACACGAAACC | CATCCGCGAGTAGGGACTTA |
| *Rab3gap2* | CACTGCCAAGGCTTTCTGTT | AACTGCTCGTAGGCCAGGT |
| *Il7r* | AAAGCATGATGTGGCCTACC | GGGAGACTAGGCCATACGAC |
| *Sidt1* | GAGACCTTCTCCACCGAAGA | TGTGGATGGCAGAGAAGATG |
| *Rnf167* | TAGTTCGTTGCATCCAGCAC | AGCTTGTCCCCATCCTCATA |
| *Sept7* | ACCAGAGGAATGCCAACAGT | ACCCCAAGGATACTGCCTTC |
| *Mapkapk3* | CCAGAAGTGTGCCCTGAAG | ATGTCCCGCATTATCTCTGC |
| *Sigirr* | CAATGGCCATCTCCCTTCTA | GAGCAGATGGGGTCCACT |
| *Herc3* | CCCAGATGTGGAAGCAATG | CCGGGATTAGGAATGTCTTC |
| *Ikbke* | GGAGGTGCTTCAGGACACG | TGCAGCCTGGTTCTTAGCTC |
| *Cep110* | AGGGAAGCTGACCGACTTCT | TAGCTGCTGCTCTGCTTTCA |
| *Fcrl1* | CTTTTGGTGAGAGGCACCTT | GAGCAGATGAGGACCAGCTT |
| *Mllt6* | GCAGCAGTGACTCCCTGAG | GCTGCTGAAGGAGGTGTCTC |
| *Rap1gds1* | TCATGGATTTGCTGGACAGA | CTGGGGGCATTTCAGATTTA |
| *D14Abb1e* | TGGAATGTATTGTAGCCTTTATGA | GGAGAAACCATCTGGAAAGGA |

**Supp Table 5.** A list of primer sequences used in validation of candidate genes with alternative splicing variants.
